# 2890. Pharmacokinetics, Safety, and Efficacy of Ceftazidime-Avibactam in Neonates and Young Infants with Bacterial Infections: Results from a Phase 2a, 2-part, Open-label, Non-randomized, Multicenter Trial

**DOI:** 10.1093/ofid/ofad500.2473

**Published:** 2023-11-27

**Authors:** John S Bradley, Emmanuel Roilides, Richard England, Margaret Tawadrous, Jean Yan, Elena Soto, Gregory Stone, Shweta Kamat

**Affiliations:** University of San Diego School of Medicine, Rady Children's Hospital, San Deigo, California; 3rd, Thessaloniki, Thessaloniki, Greece; Pfizer Inc., Groton, Groton, CT; Pfizer, Inc, Groton, Connecticut; Pfizer Inc., Sanford, Florida, USA, Sanford, Florida; Pfizer R&D UK Ltd., Canterbury, Kent, UK, Canterbury, Kent, England, United Kingdom; Pfizer, Inc., Groton, Connecticut; Pfizer India, MUMBAI, Maharashtra, India

## Abstract

**Background:**

Antibiotic resistance in pediatrics, including neonates and infants, is increasing globally. Ceftazidime-avibactam (CAZ-AVI) is approved for use in pediatric patients (age ≥ 3 months) with Gram-negative bacterial infections. This study evaluated pharmacokinetics (PK), safety, and efficacy of CAZ-AVI in hospitalized neonates and young infants (from 26 weeks gestation, up to 3 months of age) with suspected/confirmed infections due to Gram-negative pathogens requiring intravenous (IV) antibiotic treatment (Tx).

**Methods:**

This Phase 2a study was conducted at 39 sites in 9 countries (Jan2020-Dec2022; EudraCT 2018-002800-16). Hospitalized neonates and infants received single (Part A) or multiple doses (Part B) of IV CAZ-AVI (Figure 1). PK (CAZ and AVI), safety (adverse events [AEs], serious AEs, deaths), and efficacy (Part B: clinical outcomes [cure/improvement], microbiological response) were assessed. The study was not powered for inferential statistical analysis, descriptive methods were used to summarize all data.

**Figure:**
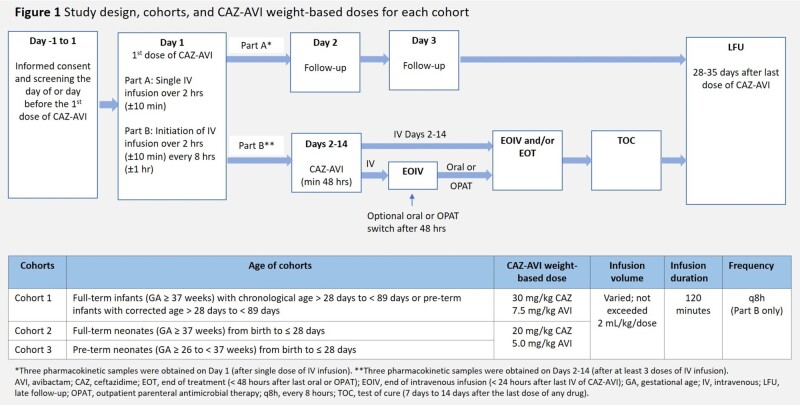

**Results:**

In total, 46 participants were administered CAZ-AVI, aged 2 to 89 days and 31 to ≥ 37 weeks gestation (Table 1). Overall, sepsis (39.1%) and urinary tract infection (15.2%) were the most common primary infectious diagnoses. In Part B, 60% of the baseline pathogens isolated were *Escherichia coli*. Median duration of CAZ-AVI Tx in Part B was 7.0 days. Plasma concentrations of CAZ and AVI were similar to those in previous pediatric studies (Figure 2). Overall, 50% (n=23) of participants had treatment-emergent AEs (TEAEs); the majority were mild/moderate in severity. SAEs (n=8) and deaths (due to necrotizing enterocolitis and septic shock, n=1; sepsis, n=1) were not related to study Tx. Most participants had favorable clinical outcomes and microbiological response at test of cure.

**Figure:**
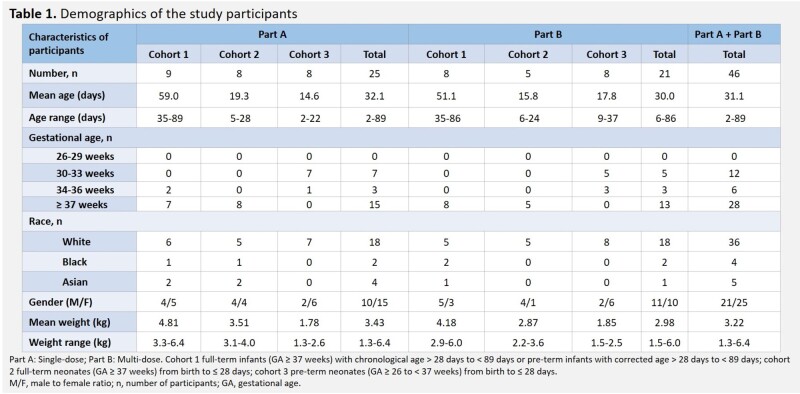

**Figure:**
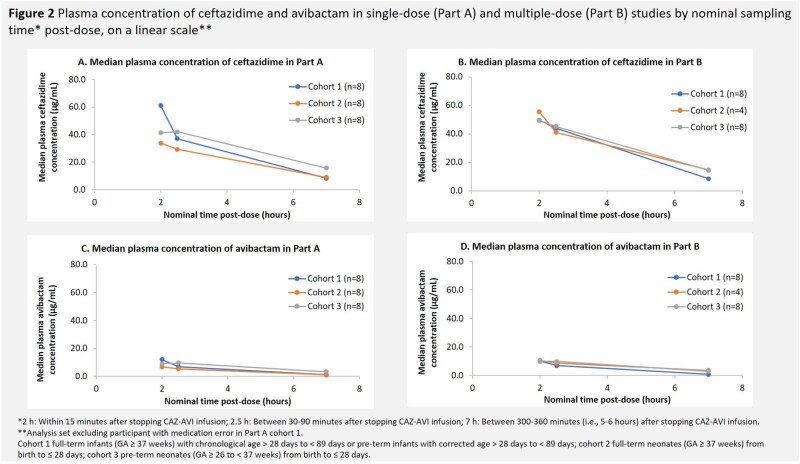

**Conclusion:**

Plasma concentrations of CAZ and AVI after single and multiple doses of CAZ-AVI were similar to previous pediatric studies. Single and multiple doses of CAZ-AVI were safe and well tolerated in hospitalized neonates and young infants with suspected/confirmed bacterial infections. TEAEs were mild/moderate in severity and no new safety concerns were identified.

**Disclosures:**

**Emmanuel Roilides, MD, PhD**, Gilead: Advisor/Consultant|GSK: Grant/Research Support|MSD: Advisor/Consultant|MSD: Grant/Research Support|Mundipharma: Advisor/Consultant|Pfizer: Grant/Research Support|Scynexis: Grant/Research Support **Richard England, MD, PhD**, Pfizer: Employee of Pfizer|Pfizer: Stocks/Bonds **Margaret Tawadrous, MD, MS**, Pfizer: Employee of Pfizer|Pfizer: Stocks/Bonds **Jean Yan, M.S.**, Pfizer: Employee of Pfizer|Pfizer: Stocks/Bonds **Elena Soto, PhD**, Pfizer: Employee of Pfizer|Pfizer: Stocks/Bonds **Gregory Stone, PhD**, Pfizer: Employee of Pfizer|Pfizer: Stocks/Bonds **Shweta Kamat, MD**, Pfizer: Employee of Pfizer|Pfizer: Stocks/Bonds

